# A study of the evacuation and allocation of hospital beds during the Covid-19 epidemic: a case study in Iran

**DOI:** 10.1186/s12913-022-08286-7

**Published:** 2022-07-05

**Authors:** F. Soroush, B. Nabilou, A. Faramarzi, H. Yusefzadeh

**Affiliations:** Department of Health Economics and Management, School of Public Health, Urmia University of Medical Sciences, Sero Street, Nazlou Paradice, Urmia, Iran

**Keywords:** Covid-19, Linear programming, Inpatients, Resource allocation, Hospital bed capacity, Epidemics

## Abstract

**Background:**

Shortage of resources, such as hospital beds, needed for health care especially in times of crisis can be a serious challenge for many countries. Currently, there is no suitable model for optimal allocation of beds in different hospital wards. The Data Envelopment Analysis method (DEA) has been used in the present study to examine the evacuation and allocation of hospital beds during the covid-19 pandemic in order to contribute to effective planning for fighting the spread the covid-19 virus.

**Methods:**

The present study was conducted in two stages in hospitals affiliated with Urmia University of Medical Sciences (UUMS) in 2021. First, the number of excess beds was determined by calculating the technical efficiency using the DEA method and Deap_2.1_ software. To reallocate excess beds to covid-19 patients, the types of hospital wards were considered. As a result of this analysis, the inefficient hospitals with excess beds in different wards, which could be used for covid-19 patients with more serious symptoms, were identified.

**Results:**

The results of the study show that the average technical efficiency of the studied hospitals was 0.603. These hospitals did not operate efficiently in 2021 and their current output can be produced with less than 61% of the used input. Also, the potential of these hospitals, over a period of 1 year, for the evacuation of beds and reallocation of them to covid-19 patients was calculated to be 1781 beds, 450 of which belonged to general wards and 1331 belonged to specialized wards.

**Conclusions:**

The DEA method can be used in the allocation of resources in hospitals. Depending on the type of hospital wards and the health condition of patients, this method can help policy-makers identify hospitals with the best potential but less emergency services for the purpose of reallocation of resources, which can help reduce the severe effects of crises on health resources.

**Supplementary Information:**

The online version contains supplementary material available at 10.1186/s12913-022-08286-7.

## Background

Covid-19, which broke out in November, 2019 and spread all over the world by March, 2020, seems to be the largest challenge facing human beings in the years following the World War II [1]. Severe Acute Respiratory Syndrome Coronavirus 2 (SARS-CoV-2) is responsible for the worldwide reported epidemic, which is spreading rapidly with more than 400 million confirmed cases and 6 million deaths [2].

Following this pandemic, the number of referrals to hospitals and those needing hospitalization rose dramatically compared to the pre-coronavirus period, necessitating the reallocation of hospital beds in different wards to covid-19 patients. As a result, only emergency patients have been admitted at most hospitals and non-emergency and elective patients have had to wait for days at times to receive health services. This, in turn, has resulted in the emergence of new problems. Since all beds in the covid-19 wards of many hospitals get quickly occupied, beds in other hospital wards should be allocated to Covid-19 patients [3, 4]. Thus, the constant change in the number of patients in different periods of the covid-19 pandemic has turned into one of the biggest challenges facing hospitals. This has made determining the optimal number of beds an important issue in the management of hospitals [5].

In general, when demand for hospital beds is unpredictable, as in the case of SARS-CoV-2, increased pressure is put on the capacity of the health system for dealing with such critical conditions. Therefore, in emergency health situations, reliable, applied models of providing information in real time situations to be used by health centers are of vital importance [6, 7]. In the case of covid-19, the challenge of the number of inpatient beds has highlighted the necessity of methods for the evaluation of performance and efficiency of hospitals in optimal utilization of hospital resources.

Non-parametric Data Envelopment Analysis method is one of the best methods that can be used to calculate the production capacity and technical efficiency of units [8, 9]. This method can be used to calculate the number of excess beds needed in hospitals in case of an increase in the number of hospitalized patients during a pandemic [10, 11].

Researchers have used different methods to study the allocation of beds in hospitals. To study the allocation of hospital beds, Kabiri et al., for example, used the Markov chain approach [12], Bahadori et al. employed the mathematical modeling method [13], and Goharani et al. utilized the business intelligence model [14]. Nepomuceno et al. used a DEA-based complexity of needs approach to study the evacuation of hospital beds during the Covid-19 pandemic [15]. DEA is a powerful non-parametric linear programming tool to estimate the technical efficiency of many service units, such as hospitals. The present study was performed via this linear programming method and taking into account the complexity of patients’ health needs for prioritizing the reduction of beds.

The present study aims to examine the evacuation and reallocation of hospital beds during the covid-19 pandemic in the hospitals affiliated with UUMS in order to develop a model for allocating hospital resources during a crisis such as covid-19.

## Methods

The present study is of the applied type. The research population included a total of 21 hospitals, affiliated with UUMS. The data for the study, collected through the Deputy of Treatment of the UUMS, included the number of physicians, nurses, other staff members, number of beds and the bed occupancy rate in the year 2021.

This study was conducted in two phases. In the first phase, using the DEA method and the Deap_2.1_ software, the technical efficiency of the hospitals and the number of excess beds, which can be reduced by hospitals to achieve higher efficiency, were calculated. In fact, in this method, the optimal number of beds can be calculated for each hospital. The mathematical equation for the DEA using the variable returns to scale and input oriented will be as follows:

$${\displaystyle \begin{array}{l}\operatorname{Min}\_\left(\uplambda, \mathrm{OS},\mathrm{IS}\right)-\left(\mathrm{M}\overset{\acute{\mkern6mu}}{1}\mathrm{OS}+\mathrm{K}\overset{\acute{\mkern6mu}}{1}\mathrm{IS}\right)\\ {}\mathrm{St}:-{\mathrm{y}}_{\mathrm{i}}+\mathrm{Y}\uplambda -\mathrm{OS}=0,\\ {}\begin{array}{l}{\uptheta}^{\ast }{\mathrm{x}}_{\mathrm{i}}-\mathrm{X}\uplambda -\mathrm{OS}=0\\ {}N\overset{\acute{\mkern6mu}}{1}\times \lambda \le 0,\lambda \ge 0, OS\ge 0, IS\ge 0\end{array}\end{array}}$$

In the above equation, the first constraint indicates that in any hospital, the excess product is zero only if (−y_i_ + Yλ) is zero. The second one signifies that the excess factors of production can be zero when (θ^∗^x_i_ − Xλ) is zero. The third constraint shows the variable returns to scale. λ is the N × 1 vector that shows the weights of the reference set. OS is the excess output vector with the dimensions M × 1 and IS is the excess input vector with dimensions K × 1, and M and K are the vectors with dimensions M × 1 and K × 1, respectively. Vector λ shows the weights that include the linear composition of the reference hospitals of the i^th^ hospital. Here, in order to calculate the value of θ^∗^ (technical efficiency) for each hospital, the linear planning should be calculated *n* times (the number of hospitals).

In this equation, other than technical efficiency, the scale efficiency and the managerial efficiency can be evaluated. The technical efficiency of a hospital is the ability of the hospital to produce a certain output using minimum input, and the scale efficiency of unit can be calculated through a comparison between the observed efficiency of that unit and efficiency at an optimal scale. In many studies, technical efficiency and variable returns to scale are divided into scale efficiency and net technical efficiency, with net technical efficiency being called managerial efficiency as well. These efficiencies are between 0 and 1, with the number being closer to 1 showing a higher efficiency [16].

In the second phase, the number of optimal beds to be reallocated in each ward is determined considering whether they are general wards (*w*_*g*_) or specialized wards (*w*_*s*_). In general, patients are hospitalized either in general wards (*w*_*g*_) or specialized wards (*w*_*s*_). General wards are dedicated to dialysis, thalassemia, emergency, oncology, gynecology, and some other beds, and specialized wards include different special care units such as ICU, CCU and isolation rooms.

The evacuation of hospital beds in special care units (${\mathrm{EV}}_{{\mathrm{w}}_{\mathrm{s}}}$) is thus calculated:

$${\mathrm{EV}}_{{\mathrm{w}}_{\mathrm{s}}}=\left\{\begin{array}{l}\mathrm{w}\left(1-{\uptheta}^{\ast}\right)-{w}_g,\kern0.5em \mathrm{w}\mathrm{hen}\ \mathrm{w}\left(1-{\uptheta}^{\ast}\right)-{\mathrm{EV}}_{{\mathrm{w}}_{\mathrm{g}}}>0,\\ {}\begin{array}{cc}0& \mathrm{otherwise}\end{array}\end{array}\right.$$

And the evacuation of beds in general wards (${\mathrm{EV}}_{{\mathrm{w}}_{\mathrm{g}}}$) is calculated as follows:

$${EV}_{w_g}=\left\{\begin{array}{l}\begin{array}{cc}{w}_g,& \mathrm{when}\ \mathrm{w}\left(1-{\uptheta}^{\ast}\right)-{w}_g\ge 0,\end{array}\\ {}\begin{array}{cc}0& \mathrm{and}\ \mathrm{otherwise}\end{array}\end{array}\right.$$

where θ^∗^ is the technical efficiency and w is the number of beds in both general and specialized wards. Based on the above equations, the optimal number of beds in specialized wards depends on the number of evacuated beds in general wards [17].

## Results

Characteristics of input and output variables to measure the efficiency of hospitals in UUMS are presented in Table 1. In total, 21 *public* hospitals affiliated to UUMS in the cities of *West Azerbaijan Province* located in *northwest Iran* with 765 physicians, 4644 Nurses, 4484 other staff, and 3288 beds were responsible for an average of 44.41% of the total bed occupancy during 2021. The highest and lowest input of beds belonged to Hospital 20, with 587 beds, and Hospital 4, with 30 beds, respectively. Also, the highest and lowest output of bed occupancy rate belonged to Hospital 1, 72.41%, and Hospital 4, 19.89%.

**Table 1:** Descriptive information about the data

Hospital	Output	Inputs			
	Bed occupancy rate (%)	Number of physicians	Number of nurses	Number of other staff	Number of beds
1	72.406	28	364	327	199
2	43.381	23	136	128	141
3	49.119	34	133	155	88
4	19.885	14	65	87	30
5	27.637	17	50	60	45
6	48.808	72	394	330	257
7	46.862	37	182	143	118
8	29.915	12	58	90	49
9	40.894	42	196	201	210
10	43.089	42	200	205	179
11	67.138	17	274	238	148
12	21.695	12	63	84	31
13	53.022	19	160	168	89
14	22.594	11	53	92	50
15	56.429	58	319	336	256
16	58.65	61	376	353	343
17	29.761	24	151	188	97
18	28.005	28	256	149	133
19	51.386	46	190	218	177
20	67.706	152	930	826	587
21	54.223	16	94	106	61
Mean	44.4097619 ± 15.88	36.42857 ± 31.74	221.14286 ± 195.87	213.5238095 ± 166.95	156.571 ± 129.82

Different kinds of efficiency in the hospitals affiliated with UUMS have been calculated using the input-oriented approach and the variable returns to scale, as presented in Table 2.

**Table 2:** Technical efficiency, managerial efficiency and scale efficiency of the hospital in the study in 2021

Hospital	Technical efficiency	Managerial efficiency	Scale efficiency	
1	0.687	1	0.687	Decreasing returns to scale
2	0.663	0.697	0.95	Increasing returns to scale
3	0.64	0.652	0.982	Increasing returns to scale
4	0.746	1	0.746	Increasing returns to scale
5	0.958	1	0.958	Increasing returns to scale
6	0.289	0.293	0.987	Increasing returns to scale
7	0.641	0.652	0.982	Increasing returns to scale
8	0.894	1	0.894	Increasing returns to scale
9	0.398	0.413	0.964	Increasing returns to scale
10	0.411	0.423	0.971	Increasing returns to scale
11	1	1	1	Constant returns to scale
12	0.787	1	0.787	Increasing returns to scale
13	0.8	0.822	0.973	Increasing returns to scale
14	0.739	1	0.739	Increasing returns to scale
15	0.328	0.391	0.84	Decreasing returns to scale
16	0.325	0.428	0.758	Decreasing returns to scale
17	0.363	0.5	0.726	Increasing returns to scale
18	0.367	0.509	0.722	Increasing returns to scale
19	0.469	0.47	0.998	Increasing returns to scale
20	0.16	0.305	0.525	Decreasing returns to scale
21	1	1	1	Constant returns to scale
Mean	0.603	0.693	0.866	

Based on the results presented in the above table the average technical efficiency of the hospitals in this study was 0.603, with Hospital 6 having the lowest technical efficiency (0.289) and Hospitals 11 and 21 having the highest technical efficiency (1). A study of the returns to scale showed that most hospitals had increasing returns to scale – 19.05% of hospitals had decreasing returns to scale (inputs should be decreased to improve efficiency), 9.5% had constant returns to scale (changes in the amount of inputs does not affect efficiency), and 71.4% had increasing efficiency (inputs should be increased to improve efficiency). The average net technical efficiency – i.e. the managerial efficiency – of the hospitals was 0.693, which means that better management and organized work can increase it by up to 30.7%. Also, eight hospitals had the maximum managerial efficiency, i.e., 1, and 13 hospitals had a managerial efficiency of less than 1. In other words, 38% of the hospitals had an inefficient management and 62% had efficient management. Hospital 6 had the lowest managerial efficiency. The average scale efficiency in these hospitals was 0.866, meaning that because of their failure to act at an optimal scale and not basing their activities on the right proportion of inputs and output, they couldn’t use 13.4% of the inputs. Of the 21 hospitals in the study, two hospitals had a scale efficiency score of 1 and constant returns to scale. The other 19 hospitals did not have good scale efficiency, with the lowest score belonging to Hospital 20.

Table 3 shows the optimal number of beds in the hospitals in the study as well as the number of beds that could be evacuated during the covid-19 epidemic. For example, hospital 1, with a technical efficiency score of 0.687, can increase efficiency by producing the same output using 31.3% (1–0.687) fewer resources. In the above table, the optimal number of beds was obtained by multiplying the technical efficiency score by the number of beds in each hospital. Then, to determine the number of beds that could be evacuated, the total number of beds was reduced from the total number of beds.

**Table 3:** Hospitals with the potential for bed evacuation

Hospital	Technical efficiency	Number of beds	Optimal number of beds	Number beds to be evacuated
1	0.687	199	137	62
2	0.663	141	93	48
3	0.64	88	56	32
4	0.746	30	22	8
5	0.958	45	43	2
6	0.289	257	74	183
7	0.641	118	76	42
8	0.894	49	44	5
9	0.398	210	84	126
10	0.411	179	74	105
11	1	148	148	0
12	0.787	31	24	7
13	0.8	89	71	18
14	0.739	50	37	13
15	0.328	256	84	172
16	0.325	343	111	232
17	0.363	97	35	62
18	0.367	133	49	84
19	0.469	177	83	94
20	0.16	587	94	493
21	1	61	61	0
Total	0.603095	3288	1500	1788

Table 4 shows the potential of the hospitals in the study for the evacuation of beds based on the type of hospital wards during the covid-19 epidemic. The results of the study indicate that, considering the inefficient hospitals, the postponement of some surgical operations and reducing the length of stay of patients in these hospitals, and by following the model of reference hospitals, a total of 1781 beds (EV_w_) could be evacuated and reallocated to covid-19 patients over a period of 1 year. Four hundred fifty of these beds are in the general wards (${\mathrm{EV}}_{{\mathrm{w}}_{\mathrm{g}}}$) and 1331 of them are in specialized wards (${\mathrm{EV}}_{{\mathrm{w}}_{\mathrm{s}}}$).

**Table 4:** The potential for the evacuation of beds in different hospitals wards

Hospital	Evacuation of beds in specialized wards (${\mathrm{EV}}_{{\mathrm{w}}_{\mathrm{s}}}$)	Evacuation of beds in general wards (${\mathrm{EV}}_{{\mathrm{w}}_{\mathrm{g}}}$)	The optimal number of Evacuated beds (EV_w_)	Monthly estimation
1	30	32	62	5
2	21	26	47	4
3	13	18	31	3
4	5	3	8	1
5	0	0	0	0
6	39	144	183	15
7	18	25	43	4
8	0	0	0	0
9	32	95	127	11
10	27	79	106	9
11	0	0	0	0
12	5	2	7	1
13	13	4	17	1
14	8	6	14	1
15	38	134	172	14
16	51	180	231	19
17	15	47	62	5
18	20	64	84	7
19	27	67	94	8
20	88	405	493	41
21	0	0	0	0
Total	450	1331	1781	149

## Discussion

The present study was carried out to examine the evacuation and reallocation of hospital beds in critical situations such as the covid-19 epidemic in order to develop a framework for the allocation of resources in hospitals during crises.

Variables such as the number of physicians, nurses and other staff members, and the number of beds were used as the input and the percentage of bed occupancy was selected as the output in the present study. The non-parametric DEA method was used in the study for the purpose of minimizing the input as in the health system managers have a greater control over the inputs rather than the output.

The technical efficiency of the hospitals affiliated with UUMS in 2021 was calculated to be 0.603. The results showed that most hospitals were not efficient and the technical efficiency could be increased by 39.7%. The hospitals with maximum technical efficiency, i.e. technical efficiency of 1, had the same original and optimal input values for their beds, with an excess bed input of zero. These hospitals had high managerial efficiency as well. However, those hospitals with technical efficiency of less than 1 had excess input beds and had to optimize the number of their beds in order to reach optimal efficiency. The results of the present study reveal that there was an excess input of beds in these hospitals, necessitating better employment of resources through more efficient management. The results of the study by Lee and Choi indicate that on average there is a 23.5% excess of beds in the hospitals of Florida [18], which is consistent with the results of the present study.

In our study, the least efficient hospital, i.e. hospital 20, which is one of the most important hospitals of the province with 493 beds for reallocation, had the technical efficiency score of 0.16.

When there is a huge demand for health services, as in the case of SARS-CoV-2 pandemic, the pressure on the health system may force hospitals to increase the capacity of their resources, such the number of hospital beds, in order to deal with such a situation. Azari et al. [19] concluded that acute respiratory distress syndrome (ARDS) is the most common clinical presentation in patients who require hospitalization in an intensive care unit. So they recommended profoundly increasing the number of ICU beds [19]. In Murray’s study [20] a significant increase in the number of hospital beds following the outbreak of covid-19 has been predicted. Therefore, in such situations, optimal utilization of hospital capacity and inputs is more efficient than building new hospitals to meet the needs of patients.

In the present study, the evacuation of beds was studied in light of the type of hospital wards. The results of the study showed that a total of 1781 bed could be evacuated in 81% of the hospitals in the study, 75% of which belonged to the specialized inpatient wards and 25% belonged to the general wards. The results of the study by Nepomuceno et al. entitled “A DEA-based complexity of needs approach for hospital beds evacuation during the covid-19 outbreak” showed that 3772 beds could be evacuated in 64% of the hospitals in their study, 82% of which being moderate complexity evacuations [15].

In the present study, hospital 5 and hospital 8, despite their potential for evacuation of beds, showed a relatively high technical efficiency, making it unnecessary for them to evacuate any beds. Most of the hospitals in the study, needed to evacuate less than 50% of their beds in specialized wards.

This method enables policy-makers to identify hospitals with highest potential and less emergency services for the purpose of evacuation and reallocation of beds and provision of resources. Among the limitations of the present study we can refer to the exclusion of the hospitals in the province not affiliated with a university. Therefore, in order to calculate the capacity for hospitalization in the province for dealing with problems such as the covid-19 pandemic all hospitals should be included in studies.

## Conclusion

The DEA method can be of great importance in the allocation of resources and improvement of the efficiency of hospitals in dealing with covid-19 based on the health conditions of patients and it can help health managers and policy-makers allocate resources more efficiently.

## Supplementary Information

**Additional file 1.**

## Data Availability

The authors confirm that the data supporting the findings of this study are available within its supplementary materials.
